# Comparative therapeutic index, lethal time and safety margin of various toxicants and snake antivenoms using newly derived and old formulas

**DOI:** 10.1186/s13104-020-05134-x

**Published:** 2020-06-16

**Authors:** Saganuwan Alhaji Saganuwan

**Affiliations:** Department of Veterinary Pharmacology and Toxicology, College of Veterinary Medicine, Federal University of Agriculture, P.M.B. 2373, Makurdi, Benue Nigeria

**Keywords:** Therapeutic index, Safety margin, Efficacy, Toxicity, Weight, Reversal, Drug

## Abstract

**Objective:**

The assessment of clinical efficacy and toxicity is very important in pharmacology and toxicology. The effects of psychostimulants (e.g. amphetamine), psychotomimetics (e.g. Cannabis sativus) and snake antivenoms are sometimes unpredictable even at lower doses, leading to serious intoxication and fatal consequences. Hence, there is need to re-assess some formulas for calculation of therapeutic index, lethal time and safety margin with a view to identifying therapeutic agents with remarkable toxicity potentials.

**Results:**

The therapeutic index formula $\left[ {T_{1} = 3\left( {W_{a} \times 10^{ - 4} } \right)} \right]$ was derived from T_1_ = LD_50_/ED_50_ and ED_50_ = $\frac{{LD_{50} }}{3} x W_{a} \times 10^{ - 4}$. Findings have shown that, therapeutic index is a function of death reversal (s), safety factor (10^−4^) and weight of animal (Wa). However, the new safety margin formula $\left[ {MS = \sqrt[3]{{\frac{{LT_{50} }}{{LD_{50} }}}} \times \frac{1}{{ED_{99} }}} \right]$ derived from LT_50_ = $\frac{{LD_{50} }}{{D_{1}^{p} }}$ and MS = $\frac{{LD_{1} }}{{ED_{99} }}$ shows that safety margin is a function of cube root of ratio between LT_50_ and LD_50_ and ED_100th_. Concentration (k) of toxicant at the receptor $\left[ {K = \sqrt[3]{{\frac{{LT_{50} }}{{LD_{50} }}}} \times \frac{1}{{T^{n} }}} \right]$ derived from D_1_ × T^n^ = K and LD_1_ = $\sqrt[3]{{\frac{{LT_{50} }}{{LD_{50} }}}}$ shows that therapeutic index, lethal time and safety margin is a function of drug or toxicant concentration at the receptor, the drug-receptor interaction and dose of toxicant or drug administered at a particular time.

## Introduction

The important assessment of clinical efficacy and toxicity of drugs and chemicals cannot be overemphasized. Dose–response relationship can identify hazardous substance [1] with toxic or beneficial effect over time [2]. Examples of such substances are snake and scorpion venoms, plant extract, drug and chemicals that cause different kinds of toxic effects on various body systems [3–11]. Attempts were made to use structures of therapeutic agents to predict their toxic effects [12, 13]. The predictive toxicity was based on active sites of compounds, such as the number of aromatic rings in polycyclic hydrocarbons, the number of chlorine atoms in chlorinated hydrocarbons and the number of hydroxyl groups. Such predictions have made some success but far from perfect [14–16]. In the past, many animals (40–100) were used for safety study until OECD introduced up-and-down procedure, limiting the number of animals for the study to 5-20 [17–19]. The use of large number of animals for determination of median lethal dose (LD_50_) has been discouraged worldwide [20]. Hence, based on the principle of R3 (Reduction, Refinement and Replacement), the number of animals for LD_50_ determination has been reduced to 2–6 animals [6]. The inherent variability, lack of predictive validity and lack of reliability of experimental animal models and conflicting clinical reports on therapeutic indices, safety margins and lethal times of some psychostimulants, psychotomimetics and snake antivenoms have necessitated the need to revise the current therapeutic index and safety margin formulas.

## Main text

### Methodology

Literatures from journals published by Elseviers, Springer, Springer Nature, Sage, Tailor and Francis, Wiley and other publishers were searched for reports on LD_50_ of amphetamine, dextroamphetamine, lysergic acid diethylamide, potassium permanganate, Abrus precatorius and tetrahydroxycannonbinol in dog, rabbit, mouse, human and rat, respectively. The d-tubocurarine has been reported to counteract their effects to some levels. However the reported LD_50_ and ED_50_ of some snake venoms and antivenoms were used for the study. The formulas used in determination of LD_50_ for snake venom with effective dose fifty (ED_50_) divided by the denominator (3) as well as other related formulas, were incorporated into derived therapeutic index, lethal time and margin safety formulas [3–27]. The derivations are as follow:

### Previously established formulas

1 $${\text{Therapeutic index }}\left( {\text{TI}} \right) = \frac{{TD_{50} }}{{ED_{50} }} = \frac{{LD_{50} }}{{ED_{50} }}$$

2 $${\text{Margin of safety }} = \frac{{TD_{1} }}{{ED_{99} }} = \frac{{LD_{1} }}{{ED_{99} }}$$

3 $${\text{Effective dose fifty }}\left( {{\text{ED}}_{ 50} } \right){\text{ for snake antivenom }} = \frac{{LD_{50} }}{3} \times {\text{Wa}} \times 10^{ - 4}$$

#### Newly derived formulas

4 $${\text{If LD}}_{ 50} = {\text{ TI}} \times {\text{ED}}_{ 50}$$

Substitute for LD_50_ in Eq. (3)

5 $$\begin{aligned} {\text{ED}}_{ 50} = \frac{{TI \times ED_{50} }}{3} \times {\text{Wa}} \times 10^{ - 4} \hfill \\ = \frac{{\frac{{TI \times ED_{50} }}{3}}}{{ED_{50} }} = {\text{ Wa}} \times 10^{ - 4} \hfill \\ \end{aligned}$$

6 $$= \frac{{TI \times ED_{50} }}{3} \times \frac{1}{{ED_{50} }} = {\text{ Wa}} \times 10^{ - 4}$$

7 $$= \frac{TI}{3} = {\text{ Wa}} \times 10^{ - 4}$$

8 $$= {\text{ TI }} = { 3}({\text{Wa}} \times 10^{ - 4} )$$

TI = Therapeutic index; LD_50_ = Median lethal dose; ED_50_ = Median effective dose; Wa = Weight of animal; 10^−4^ = Safety factor.

#### Integration of lethal time with margin safety formula

9 $${\text{Median lethal time }}\left( {{\text{LT}}_{ 50} } \right) \, = \frac{{LD_{50} }}{{D^{p} }}$$

10 $${\text{LD}}_{ 50} = \frac{{LT_{50} }}{{LD_{1}^{p} }}$$

whereas LD_1_ = Dose that kills one animal; p = Exponent (1/3).

Remember Eq. (2) for margin of safety (MS) = $\frac{{LD_{1} }}{{ED_{99} }}$.

11 $${\text{LD}}_{ 1} = {\text{ MS}} \times {\text{ED}}_{ 9 9}$$

12 $$LD_{1}^{P} = \frac{{LT_{50} }}{{LD_{50} }}$$

But p = 1/3.

Therefore

13 $$LD_{1}^{{\frac{1}{3}}} = \frac{{LT_{50} }}{{LD_{50} }}$$

14 $${\text{LD}}_{ 1} = \sqrt[3]{{\frac{{LT_{50} }}{{LD_{50} }}}}$$

So, integrate Eqs. (11) and (14)

15 $${\text{LD}}_{ 1} = {\text{ MS}} \times {\text{ED}}_{ 9 9} = \sqrt[3]{{\frac{{LT_{50} }}{{LD_{50} }}}}$$

Hence,

16 $${\text{MS }} = \sqrt[3]{{\frac{{LT_{50} }}{{LD_{50} }}}} \times \frac{1}{{ED_{99} }}$$

Therefore, margin of safety is a function of cube root of ratio between LT_50_ and LD_50_ and one-hundredth of ED.

#### Integration of time of exposure with toxic or lethal dose

17 $${\text{Concentration of toxicant }}\left( {\text{K}} \right) \, = {\text{ D}} \times {\text{T}}^{\text{n}}$$

whereas D = Daily dose; T = Time of exposure; K = constant which is the concentration of toxicant causing toxicity; n = power of exponent.

Therefore,

18 $${\text{D }} = \frac{{T^{n} }}{K}$$

But if D can kill one animal as shown in Eq. (15) and related to Eq. (18), it would be referred to as TD_1_

19 $$\therefore {\text{LD}}_{ 1} = {\text{ TD}}_{ 1} = \sqrt[3]{{\frac{{LT_{50} }}{{LD_{50} }}}} = \frac{{T^{n} }}{K}$$

Therefore,

20 $${\text{K }} = \sqrt[3]{{\frac{{LT_{50} }}{{LD_{50} }}}} \times \frac{1}{{T^{n} }}$$

The formulas were used to calculate LD_50_, ED_50_, LT_50_, LD_1_, ED_99_, therapeutic index (TI) and safety of margin for all the reported antidotes for snake envenomation, *Abrus precatorius*, lysergic acid diethylamide, tetrahydroxycannabinol, amphetamine, methamphetamine, dextroamphetamine and potassium permanganate poisoning. All the LT_50_ in hour and minute should be converted to second.

### Results

The LD_50_, ED_50_, LT_50_, LD_1_, ED_99_, dose of toxicants, therapeutic index and safety margin of amphetamine, dextroamphetamine, methamphetamine, lysergic acid diethylamide, tetrahydroxycannabinol, potassium permanganate and *Abrus precatorius* are presented in Table 1.The LD_50_, ED_50_, LD_1_, ED_99_, therapeutic index and safety margin of snake venoms and antivenoms are presented in Table 2.

**Table 1:** Therapeutic indices and safety margins of some chemicals and plant extract

Toxicant	Experimental		Weight of animal(kg)	LD_50_ (mg/kg)	ED_50_ (mg/kg)	LT_50_	LD_1_ (mg/kg)	ED_99_ (mg/kg)	T_1_ (Control)	T_2_	MS_1_ (Control)	MS_2_
	Animal	Antidote										
Amphetamine	Dog	d-Tubocucarine	10	5.9 i.v	2.0	2.9 s	0.118	3.96	2.95	3.0	0.03	0.13
Dextroamphetamine	Dog	d-Tubocucarine	10	11.8 i.v	3.9	7.3 s	0.236	7.72	3.02	3.0	0.03	0.11
Methamphetamine	Dog	d-Tubocucarine	10	11.8 i.v	3.9	7.3 s	0.236	7.72	3.02	3.0	0.03	0.11
Lysergic acid diethylamide	Rabbit	d-Tubocucarine	1.8	0.3 i.v	0.02	0.6 s	0.006	0.04	15.0	0.54	0.15	13.2
Potassium permanganate	Mouse	d-Tubocucarine	0.02	1499.7 oral	1.0	1.3 h	30.0	1.98	1499.7	0.003	15.1	1.58
*Abrus precatorius*	Human	d-Tubocucarine	60	197 i.m	394	5.2 min	3.94	780.1	0.5	18.0	0.005	0.002
Tetrahydroxy cannabinol	Rat	d-Tubocucarine	0.15	29 i.v	0.15	24.2 min	0.58	0.30	193.3	0.05	1.9	12.2

**Table 2:** Therapeutic indices and safety margins of the antidotes of some snake venoms

Species	LT_50_ (hr)	LD_50_ (mg/kg)	ED_50_ (mg/kg)	LD_1_ (mg/kg)	ED_1_ (mg/kg)	ED_99_ (mg/kg)	T_2_	MS_1_ (Control)
*Crotalus durissus terrificus* (Tropical rattle snake)	0.43	0.13	4.02	0.026	0.0804	7.96	0.69	0.003
*Crotalus scutulatus scutulatus* (Mojave rattle snake)	0.52	0.17	4.40	0.034	0.088	8.71	0.85	0.004
*Crotalus horridus africaudatus* (Canebrake rattle snake)	1.61	0.92	7.72	0.184	0.1544	15.29	4.60	0.012
*Crotalus adamanteus* (Eastern diamond back rattle snake)	2.08	1.35	8.77	0.27	0.1754	17.36	6.75	0.016
*Crotalus durissus durissus* (Central American rattle snake)	2.51	1.79	9.64	0.358	0.1928	19.09	8.95	0.019
*Agkistrodon piscivorus piscivorus* (Eastern cotton mouth)	3.84	3.38	11.91	0.676	0.2382	23.58	16.90	0.029
*Croatlus viridus helleri* (Southern pacific rattle snake)	3.92	3.48	12.03	0.696	0.2406	23.82	17.40	0.029
*Crotalus molossus molossus* (Northern black-tailed rattle snake)	4.17	4.42	13.03	0.884	0.2606	25.80	22.10	0.034
*Sistrurus miliarius barbourin* (Southern pygmy rattle snake)	4.91	4.87	13.45	0.974	0.269	13.45	24.35	0.072
*Agkistrodon contortrix contortrix* (Southern Copperhead)	5.0	4.99	13.56	0.998	0.2712	13.56	24.95	0.073
*Crotalus horridus horridus* (Timber rattle snake)	5.85	6.32	14.61	1.264	0.2922	14.61	31.60	0.087
*Micrarus fulvius*	0.79	0.32	4.77	0.064	0.0954	9.44		0.007

Consroe et al. established baseline LD_50_ values for crotalid anti venin FAB prepared from sheep immune globulin (IgG)

### Discussion

Side effects, adverse drug reactions, untoward effects, side toxicity and idiosyncratic effects associated with drugs may be due to normal dose, under dose or drug over dose [5, 28]. The calculated therapeutic index of amphetamine (2.95), dextroamphetamine and amphetamine (3.02) using the previously established formula as compared to therapeutic index of 3.0 for the three drugs using the new formula show that, the newly developed formula can be used for calculation of therapeutic index of some psychomimetic and psychotomimetic drugs. However, the previously established formula yielded very high therapeutic index for LSD (15.0), potassium permanganate (1499.7), *Abrus precatorius* extract (0.5) and tetrahydroxycannabinol (193.3) as compared to 0.54, 0.003, 18.0 and 0.05 yielded by the newly developed formula, respectively. The findings agree with the report indicating that the conventional formula for calculation of therapeutic index is not a truthful measure of safety of a drug in clinical setting [10]. The low therapeutic index of 0.05 for tetrahydroxycannabinol agrees with the report that most biologically active molecules of *Cannabis sativa* have no therapeutic uses [24]. Very low therapeutic index (0.003) of potassium permanganate yielded by the newly derived formula agrees with the report indicating that the chemical is highly toxic [4]. The associated toxicity signs are rapid shallow respiration, diarrhea, gastroenteritis, liver and kidney damage and death.

The low therapeutic index (0.5) of* A.precatorius* shows that the plant is very toxic. This may be due to presence of toxic principle called abrin [29]. However, the relatively high therapeutic index of 18.0 calculated using the new formula agrees with the report that the plant may have some degrees of therapeutic safety [21]. The therapeutic index for LSD using the conventional (15) and new formula (0.54) corroborates the findings that the pharmacology of LSD is complex and its mechanism of actions is not understood [25]. *A. precatorius* extract is more toxic when given intraperitoneally as compared to oral route [11]. However d-tubocurarine can alleviate toxicity effects of amphetamine, dextroamphetamine, methamphetamine [26], *Abrus precatorius* [30], tetrahydroxycannabinol [31], potassium permanganate [32], and lysergic acid diethylamide [33].

The dose-toxicity response pattern in graded fashion may culminate in LD_50_ and could be counteracted by therapeutic dose 50. This explains individual variation of susceptibility to doses of toxicants as proven by low therapeutic index (0.0007) of *Micrarus fulvius* antivenom (Table 2) as compared to high toxicity potential of *M. fulvius* venom [11]. The low to high therapeutic indices of all the snake antivenoms in the present study indicate that, treatments of snake envenomation is by toxin neutralization, using specific antidotes for specific snakes [8]. The obtaining of therapeutic index (0.006–1499.7) in the present study disagrees with the report of Stanley indicating that therapeutic index could be 33,000:1 [34]. Therefore one vial of the relevant antivenom is sufficient for the circulating venom, but recovery time may be delayed, because many clinical and laboratory effects are not reversed immediately [35]. Hence clinical trials of antivenoms are potentially more important and useful [36]. Pain score of more than two (2) requires additional antivenom and patient should be frequently assessed [37] for improvement. Therefore, there is need for clinicians and laboratory toxicologists to improve therapeutic knowledge of snake envenomation [38]. Cardio-respiratory distress, coagulopathy and swelling in the first hours of admission are poor prognostic signs associated with weak therapeutic response to snake envenomation [39]. Effective dose 99 (7.96–23 mg/kg) agrees with the report indicating that, there are many recommended therapeutic interventions, which are ineffective and may be harmful [40]. Therefore, more purified and specific antivenoms are required to avoid post-treatment reactions [41], suggesting that polyvalent antivenom may be less effective against neurotoxic snake bite [42], translating to 1:2 required 30 vials of antivenom [43]. Paraspecific neutralization of snake venom by antivenom could induce coagulopathy in the affected patients [44]. Efficient, safety and thermal stability have been reported for freeze-dried trivalent antivenom for snake bites in larger phase III trial [45]. Russell’s viper injects 63-70 mg of venom during the first bite and each vial of polyvalent antivenom neutralizes 6 mg of the venom, 8-10 vials are required in majority of the cases [46]. Neither antivenom nor time of its administration affects venom-induced coagulopathy [35]. Low dose of 20–220 ml reduced the hospital stay as compared to 40–550 ml dose, suggesting that the lower the dose of snake antivenom the more effective the antivenom. Fatality rates of 15.4% and 17.6% for 2 and 4 vials of antivenom as compared 223% have been reported [47]. Protection of snake antivenom against *Echis ocellatus* is 21–99% in Nigeria [36]. Hence, the number of animals for similar study can be reduced [5]. The LT_50_ (0.065–24.2 min) of all the animal, plant and chemical toxins in the present study shows the importance of dose-time-response relationship in identification of hazards [1].

## Conclusion

The newly derived formulas yielded low and safer values for therapeutic indices and standard safety margins of drugs, toxicants, venoms, antivenom and other xenobiotics. But the safety of therapeutic agent is dependent on dose, lethal time, body weight, frequency and time of administration and safety factor of the drug.

## Limitations

The calculations were based on the findings from experiments conducted in various laboratories across the globe. All the lethal times have to be converted to seconds. The derived formulas were applied on different species of toxic animals and plants.

## Data Availability

All data generated or analyzed during this study are included in this published article.

## Abbreviations

LT_50_: Median lethal time
LD_50_: Median lethal dose
ED_50_: Median effective dose
LD_1_: Lethal dose per one animal
ED_1_: Effective dose per one animal
ED_99_: Effective dose per 99 animal
LD_1_: Lethal dose 1
T_1_: Therapeutic index for the old formula
T_2_: Therapeutic index for the new formula
MS_1_: Margin of safety for new formula
MS_2_: Margin of safety for the new formula
